# Reactive Species from Two-Signal Activated Macrophages Interfere with Their Oxygen Consumption Measurements

**DOI:** 10.3390/antiox10071149

**Published:** 2021-07-20

**Authors:** Panagiotis F. Christopoulos, Mantas Grigalavicius, Alexandre Corthay, Kristian Berg, Theodossis A. Theodossiou

**Affiliations:** 1 Tumor Immunology Lab, Department of Pathology, Rikshospitalet, Oslo University Hospital and University of Oslo, 0424 Oslo, Norway; alexandre.corthay@ous-research.no; 2 Department of Radiation Biology, Institute for Cancer Research, Oslo University Hospital, 0379 Oslo, Norway; Mantas.Grigalavicius@rr-research.no (M.G.); kristian.berg@rr-research.no (K.B.)

**Keywords:** nitric oxide (NO^●^), superoxide anion (O_2_^●−^), classically activated macrophages (M1), reactive oxygen/nitrogen species (ROS/RNS), respiration, oxygen consumption

## Abstract

Metabolic modulation of macrophage activation has emerged as a promising strategy lately in immunotherapeutics. However, macrophages have a broad spectrum of functions and thus, understanding the exact metabolic changes that drive a particular immune response, is of major importance. In our previous work, we have reported a key role of nitric oxide (NO^●^) in two(2)-signal activated macrophages [M(2-signals)]. Further characterization using metabolic analysis in intact cells, showed that the basal and maximal respiration levels of M(2-signals) were comparable, with cells being unresponsive to the injections-inducd mitochondrial stress. Here, we show that excessive NO^●^ secretion by the M(2-signals) macrophages, interferes with the oxygen (O_2_) consumption measurements on cells using the seahorse metabolic analyzer. This is attributed mainly to the consumption of ambient oxygen by NO^●^ to form NO_2_^−^ and/or NO_3_^−^ but also to the reduction of O_2_ to superoxide anion (O_2_^●−^) by stray electrons from the electron transport chain, leading to the formation of peroxynitrite (ONOO^−^). We found that reactive species-donors in the absence of cells, produce comparable oxygen consumption rates (OCR) with M(2-signals) macrophages. Furthermore, inhibition of NO^●^ production, partly recovered the respiration of activated macrophages, while external addition of NO^●^ in non-activated macrophages downregulated their OCR levels. Our findings are crucial for the accurate metabolic characterization of cells, especially in cases where reactive nitrogen or oxygen species are produced in excess.

## 1. Introduction

Immunometabolism has, as of late, emerged as a highly promising research field, in various immune-related human pathologies including cancer [1]. In contrast to most immune cells, macrophages have a diverse role in cancer, as well as in autoimmune diseases progression or regression, depending on their activation status (M1 or M2). As such, metabolic manipulation of macrophages has been suggested to hold a great potential for clinical applications [2]. Given the complexity of the field and the fact that immune cells perform also non-immune related functions, understanding the exact metabolic changes that drive a particular immune response is of major importance. 

Similar to the Warburg effect in cancer cells, it has been reported that some immune cells like T cells and/or macrophages exhibit a downregulated oxidative phosporylation (OXPHOS) and hence rely on glycolysis following their activation [3,4]. A hallmark of classically activated murine macrophages (M1) is the production of nitric oxide (NO^●^) [5]. We have recently reported a key role for NO^●^ in the tumoricidal activation of two(2)-signals activated macrophages [6]. Interestingly, further metabolic characterization of these anti-tumor macrophages [M(2-signals)] in intact cells, using the Seahorse metabolic analyzer, revealed that these macrophage subpopulations seemed completely unresponsive to injections of several OXPHOS modulators like oligomycin, FCCP and myxothiazol, with the basal oxygen consumption levels (media only) being equal to the maximal (following FCCP administration). Furthermore the basal respiration of these 2-signal activated macrophages seemed to be systematically divergent from the other subgroups and control macrophages (unpublished data). These observations led to us to hypothesize that the excessive NO^●^ secretion by the M(2-signals) macrophages, as well as the O_2_^●−^ production from the reduction of molecular oxygen by the electron leakage of the impaired electron transport chain, may interfere with the oxygen (O_2_) consumption measurements in Seahorse metabolic assays.

In this study, we aimed to investigate the role of NO^●^ and O_2_^●−^ in oxygen consumption, during metabolic assays in intact cells. NO^●^ is a very reactive free radical which is generated from L-arginine by the nitric oxide synthases (endothelial—eNOS, neuronal—nNOS and inducible—iNOS) [7]. In the case of M(2-signals) macrophages, the NO^●^ production is due to the upregulation of the iNOS following the treatment [8]. The inducible NO^●^ synthase is selectively inhibited by the N-([3-(Aminomethyl)phenyl]methyl)ethanimidamide (1400 W), at a selectivity ratio >5000 versus eNOS [9]. Oxygen superoxide anion (O_2_^●−^), is a product of one-electron reduction of O_2_, and is a ROS commonly produced in the cell mitochondria, due to electron leakage from the electron transport chain [10,11]. Superoxide is very reactive towards NO^●^ leading to the formation of peroxynitrire (ONOO^−^) at the astonishing rate of 6.7 × 10^9^ M^−1^ s^−1^ [12]. Moreover, NO^●^, uses oxygen from the environment to form nitrite (NO_2_¯) and nitrate (NO_3_¯) [13]. The conversion of NO^●^ either to ONOO^−^, or to NO_2_¯ and NO_3_¯, takes up a substantial amount of the ambient oxygen and therefore masks the oxygen consumption of cells producing NO^●^ and O_2_^●−^, as we show in the present work.

## 2. Materials and Methods

### 2.1. Cell Cultures and Treatments

For generation of primary bone marrow-derived macrophages (BMDMs), the femur and tibiae of the hind legs of 8–12 week-old C57/BL-6 (Janvier Labs, Le Genest-Saint-Isle, France) mice (male and female) were used as a source of hematopoietic stem cells according to the protocol previously published [6]. Animals were bred in-house at the Department of Comparative Medicine, Oslo University Hospital, Rikshospitalet, and euthanized by CO_2_ or cervical dislocation. The study was approved by the Norwegian National Committee for Animal Experiments (approval number 20/102031). For the activation of differentiated macrophages, the following conditions were used for 24 h in the indicated final concentrations in RPMI-1640 supplemented with 10% FBS and either 10% L929 (#400260, CLS Cell Lines Service, Eppelheim, Germany) conditioned medium or 10 ng/mL mouse M-CSF (#315-02, Peprotech, Rocky Hill, NJ, USA): 40 ng/mL IFNγ (#315-05, Peprotech) plus 10 ng/mL LPS (#L4391, Sigma-Aldrich, St. Luis, MO, USA) or 100 ng/mL Pam3CSK4 (#tlrl-pms, Invivogen, San Diego, CA, USA). For the ROS/NOS inhibition experiments, 50 μΜ N-([3-(Aminomethyl)phenyl]methyl)ethanimidamide (1400 W dihydrochloride, #W4262, Sigma-Aldrich), 100 μΜ carboxyPTiO potassium salt (#C221, Sigma-Aldrich), 15 μΜ Mn(III)tetrakis(1-methyl-4-pyridyl)porphyrin (ΜnTmPyP, #475872, Sigma-Aldrich) or 250 μΜ uric acid were added to macrophages for 1 h at 37 °C, prior to activation. For the NO^●^ donors experiments, 3 mM 3-Morpholinosydnonimine (SIN-1 HCl, #567028 Sigma-Aldrich) or 3 mM S-Nitroso-N-acetyl-DL-penicillamine (SNAP #N3398, Sigma-Aldrich) were added to cell-free wells or to untreated macrophages as indicated.

### 2.2. Nitric Oxide Measurements

Following the 24 h BMDM activation 50 μL of cell supernatant was mixed 1:1 with Reagent A; 1% sulphanilamide (#S9251, Sigma-184 Aldrich) and 5% phosphoric acid (#W290017, Sigma-Aldrich) in dH_2_O and incubated at room temperature in the dark for 10 min. Another 50 μL of Reagent B; 0.1% N-(1-napthyl) ethylenediamine (#N9125, Sigma-Aldrich) in dH_2_O was added. Serial dilutions of 100 μM NaNO_2_ were used for the standard curve. Absorbance at 540 nm was measured in a luminometer (BioTek Instruments, Winooski, VT, USA). For the quantification of active NO^●^, 10 mM of 4-Amino-5-methylamino-2′,7′-difluorescein (DAF-FM, #D1821, Sigma-Aldrich) was added in a Seahorse mirror plate and fluorescence measurements were taken every 5 min (λ_ex_ = 490 nm, λ_em_ = 530 nm), using a Tecan, Spark M10 plate reader (Tecan, Männedorf, Switzerland) at 37 °C.

### 2.3. Metabolic Assays

For the metabolic assays, using the XFe96 metabolic analyzer (Seahorse, Agilent Technologies, Santa Clara, CA, USA) assay, 7 × 10^4^ BMDMs were plated in Seahorse XFe96 plates (V3-PS, TC treated, Agilent Technologies) and treated with the indicated activating stimuli for 24 h. Following activation, cell media was changed to XF base medium minimal DMEM (Agilent) supplemented with 10 mM glucose, 2 mM Lglutamine, 2 mM sodium pyruvate, pH adjusted to 7.4, and incubated at 37 °C, 0% CO_2_ for 1 h prior to the metabolic assay. Cells were then subjected to a “mitostess” test consisting of Oligomycin (2 μΜ), FCCP (1 μM) and Rotentone/Antimycin A (2/2 μΜ) sequential injections (3 measurement cycles each), following the initial basal measurements (media-only, 3 or 4 measurement cycles). In all cases, both the oxygen consumption rates (OCR) and extracellular acidification rates (ECAR) were determined. The background OCR and ECAR were obtained from wells without cells (medium only), and subtracted automatically by the XFe96 software.

### 2.4. Statistics

Statistical analyses were performed using GraphPad Prism v7.04 software (GraphPad). All the central tendencies represent mean ± STD. The *p* values were generated using two-tailed Mann-Whitney U tests or non-parametric multiple comparisons with two-stage False Discovery Rate (FDR) correction of Benjamini, Krieger and Yekutieli, or Dunn’s correction, where appropriate. * indicates *p* < 0.05, ** indicates *p* < 0.01, *** indicates *p* < 0.001.

## 3. Results

### 3.1. NO^●^ and O_2_^●−^ Donors Mimic the Oxygen Consumption Profile of 2-Signal Activated Macrophages

**Figure 1:**
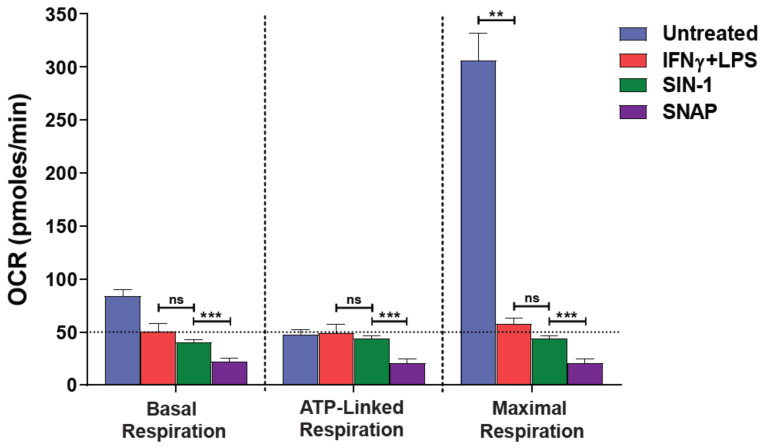
NO^●^ and O_2_^●−^ donors mimic the respiration profile of 2-signal activated macrophages. Macrophages were activated for 24 h with 40 ng/mL IFNγ plus 10 ng/mL LPS or not (untreated) and subjected to serial injections of 2 μΜ oligomycin (ATP production), 1 μΜ FCCP (Maximal respiration) and 2/2 μΜ antimycin A/rotentone, according to the “mitostress” protocol. A Seahorse XFe96 metabolic analyzer was used to record the changes in the oxygen consumption rate (OCR). The reactive species-donors SIN-1 (NO^●^ and O_2_^●−^) or SNAP (NO^●^) were added, at the concentration of 3 mM each, prior to the assay in cell-free wells. The last OCR measurement in sequence before addition of oligomycin was used to represent the basal respiration. Two-tailed Mann-Whitney U tests or the Kruskal-Wallis test (with the Benjamini-Krieger-Yekutieli two-stage linear step-up, or with the Dunn’s, procedure) were used for comparisons. ns: not significant, ** *p* < 0.01, *** *p* < 0.001. Horizontal intermittent line indicate the basal OCR levels of IFNγ + LPS activated macrophages. One representative experiment is shown, with data presented as mean ± STD of at least three replicates per condition.

**Figure 2:**
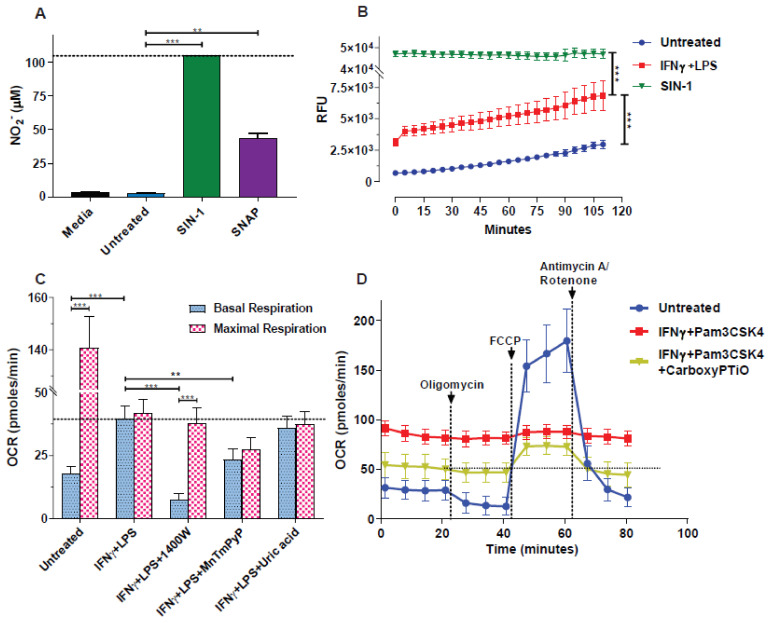
Quantification of macrophage- or donor-derived NO^●^, and the partial cell-respiration rescue by NO^●^ and O_2_^●−^ blockade. (**A**,**B**) Macrophages were activated for 24 h with IFNγ (40 ng/mL) plus LPS (10 ng/mL) or not (untreated) and cell supernatants were analyzed for (**A**) nitrites (NO_2_¯), using the Griess assay. NO_2_¯ levels were expressed as μΜ using serial dilutions of NaNO_2_ for the standard curve. Cell-free media, similar to the one used for background subtraction in Seahorse served as an additional control. (**B**) For the DAF-FM assay, serial fluorescent measurements of active NO^●^ were taken every 5 min in a Seahorse mirror plate. Values in media from cell-free wells (blank), were subtracted. (**A**,**B**) In both cases the reactive species-donors SNAP (3 mM) and/or SIN-1 (3 mM) were added to cell-free wells prior to the respective assays. The Kruskal-Wallis test (with the Benjamini-Krieger-Yekutieli two-stage linear step-up, or with the Dunn’s, procedure) was used for comparisons. ** *p* < 0.01, *** *p* < 0.001. (**C**,**D**) The ROS/RNS inhibitors/scavengers 1400 W (50 μΜ), MnTmPyP (15 μΜ), uric acid (250 μΜ) or carboxyPTiO (100 μΜ) were used as indicated to treat macrophages for 1 h prior to activation with (**C**) IFNγ + LPS (40 ng/mL and 10 ng/mL respectively) or (**D**) IFNγ + Pam3CSK4 (40 ng/mL and 100 ng/mL respectively). (**C**) The last OCR measurements in sequence before addition of oligomycin or after addition of FCCP were used to represent the basal respiration, while the highest point was used for the representation of the maximal respiration. Two-tailed Mann-Whitney U tests or the Kruskal-Wallis test (with the Benjamini-Krieger-Yekutieli two-stage linear step-up procedure) were used for comparisons. ** *p* < 0.01, *** *p* < 0.001. (**A**) Horizontal intermittent lines indicate either the measured nitrites levels of SIN-1 (assay’s plateau), (**C**) the basal OCR levels of IFNγ + LPS activated macrophages or (**D**) macrophages treated with IFNγ + Pam3CSK4 + CarboxyPTiO. Vertical intermittent lines indicate the injection points. One representative experiment is shown, with data presented as mean ± STD of at least three replicates per condition.

A hallmark of 2-signal activated macrophages [M(2-signals)] is the excessive production of NO^●^ amongst other reactive species. To explore whether the generation of RNS/ROS interfere with oxygen consumption rates (OCR) in our intact cell XFe96 metabolic assays, we employed two extracellular NO^●^ donors: SNAP and SIN-1 (the latter also produces O_2_^●−^) in media-only wells (without cells) in the templates of our “Seahorse” mitostress assay. Of note, both NO^●^ donors showed considerable oxygen consumption in the absence of cells. More importantly, the basal and maximal levels of SIN-1 were comparable to those of IFNγ + LPS pre-activated macrophages [M(2-signals)] (Figure 1). It must be noted that the non-activated macrophages exhibited a high maximal respiration upon uncoupling of OXPHOS from the ETC by FCCP. This high consumption collapses upon macrophage 2-signal activation e.g., by IFNγ + LPS (*p* < 0.01, Figure 1). As expected, the extracellular acidification levels (glycolysis) remained at background levels throughout the assays, and irrespective of oligomycin addition in the NO^●^ donors cell-free wells (ECAR—Figure S1). To confirm that both donors successfully produced NO^●^ during our experiments, we performed the Griess assay to quantify the nitrite (indirect NO^●^ measurement) accumulation (Figure 2A). Since the Griess assay is also limited in its detection range i.e., by NaNO_2_ dilutions and absorbance values, we also stratified a real-time assay measuring the florescence of DAF-FM, which is directly proportional to the active NO^●^ produced, though relative measurements can only be obtained. The experimental conditions were adjusted to mirror the Seahorse assay, in duplicate plates. Using the DAF-FM assay we found that 3 mM SIN-1 produced ~10 fold more NO^●^ than that by M(2-signals) macrophages (*p* < 0.001, Figure 2B). Thus NO^●^ and O_2_^●−^ donors assayed extracellularly, consume considerable amounts of ambient oxygen, using Seahorse.

### 3.2. Blockade of NO^●^ and O_2_^●−^ Partly Rescues the Respiration of M(2-Signals)

Following our initial observations from IFNγ + LPS pre-activated, NO^●^ producing macrophages, but also from NO^●^ donors in extracellular assays, we aimed to explore whether inhibition of NO^●^ and O_2_^●−^ could unveil the actual oxygen consumption of the activated macrophages. To this end we employed various inhibitors including: the selective iNOS (1400 W), a superoxide anion scavenger (MnTmPyP), and the peroxynitrite scavenger (uric acid), prior to, and during macrophage activation by IFNγ + LPS. Using the Seahorse metabolic analyzer, we found that 1400 W inhibition of iNOS and consequent inhibition of downstream NO^●^ production, lowered the basal respiration levels (*p* < 0.001) and restored the responsiveness of activated cells to FCCP injection (*p* < 0.001). This resulted in basal and maximal respiration, resembling more those of naïve (untreated) cells, although not reinstated to the full extent (Figure 2C). The O_2_^●−^ scavenger MnTmPyP had a lesser, yet tangible effect (than 1400 W), in restoring the basal respiration of IFNγ + LPS activated macrophages (*p* < 0.01), indicating a role of superoxide in extracellular oxygen consumption. Uric acid yielded no noticeable effect on the OCR of M(2-signals) macrophages suggesting no role of peroxynitrite removal from the system (Figure 2C). We repeated the above experiment with a different 2-signal activating stimulus (IFNγ + Pam3CSK4) and with the use of an NO^●^ scavenger (carboxyPTiO) this time. As can be seen from the results in Figure 2D, the addition of carboxyPTiO, lowers the basal respiration, restores the sensitivity of activated macrophages to the mitostress stimuli and increases the maximal respiratory capacity of the 2-signal primed cells (Figure 2D). Furthermore, using the same NO^●^ donors as before (SIN-1 and SNAP), in the presence or absence of the superoxide scavenger MnTmPyP (cell-free wells) and in accordance to our expectations, we found that upon addition of MnTmPyP to the SIN-1 solution (NO^●^ and O_2_^●−^ donor), the OCR levels dropped by ~40%, but not in the SNAP (NO^●^ only donor) solution (Figure S2). Thus, blocking/scavenging of NO^●^ and O_2_^●−^, rescues the respiration of 2-signal activated macrophages.

### 3.3. NO^●^ and O_2_^●−^ Donors Immediately Inhibit the Respiration of Naïve Macrophages

**Figure 3:**
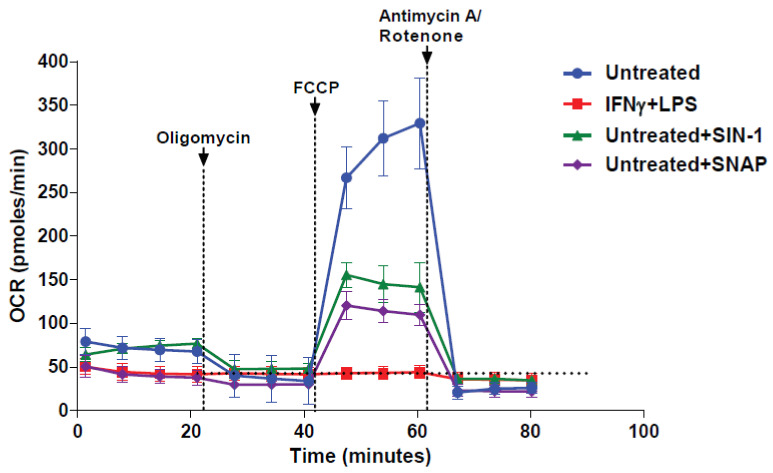
NO^●^ and O_2_^●−^ donors inhibit the respiration of naïve macrophages. Macrophages were activated for 24 h with the IFNγ + LPS (40 ng/mL and 10 ng/mL respectively) or not (untreated) and subjected to serial injections of oligomycin (2 μΜ), FCCP (1 μΜ) and antimycin A/rotentone (2/2 μΜ), according to the mitostress protocol. A Seahorse XFe96 metabolic analyzer was used to record the changes in the oxygen consumption rate (OCR). The reactive species-donors SIN-1 (NO^●^ and O_2_^●−^) or SNAP (NO^●^) were added (3 mM for either) to untreated (naïve) macrophages prior to the assay. For comparison reasons horizontal intermittent line indicate the basal OCR levels of IFNγ + LPS activated macrophages. The vertical intermittent lines indicate the injection points. One representative experiment is shown, with data presented as mean ±STD of at least three replicates per condition.

Following our observations delineated above, we sought to explore the effect of the addition of NO^●^ in naïve (untreated) macrophages by adding the NO^●^ donors SIN-1 and SNAP, just prior to the seahorse metabolic assay. We found that both NO^●^ donors hampered the oxygen consumption of naïve macrophages compared to controls, however the basal OCR was only lowered in the case of SNAP addition. It must be noted that in the case of the NO^●^ donors addition, cells were still responsive to the ECT inhibitors injections in contrast to 24 h pre-activated macrophages (IFNγ + LPS) (Figure 3) and the cell-free NO^●^ donors profile shown above (Figure 1). This indicated that NO^●^ and O_2_^●−^ have an immediate and direct effect in the cellular OCR levels.

## 4. Discussion

We have recently reported that two simultaneous signals are required for efficient anti-tumor activation of macrophages in vitro [6,8] and in vivo (unpublished data). Production of NO^●^ by these tumoricidal macrophages [M(2-signals)], was shown to possess a key role in their cytotoxic properties since blockade of iNOS, partly restored the macrophage-induced growth inhibition of cancer cells [6]. It has been previously reported that classically activated macrophages (M1) are characterized by inhibition of their respiration [3], most probably due to breaks in their Crebs cycle [14]. Interestingly, NO^●^ seems to play a major role in the mitochondrial dysfunction of these macrophages and it was postulated that IFNγ + LPS activation completely suppressed the activity of ETC complexes I and III, while partially that of complexes II and IV [14]. In fact it is well documented that the only source of oxygen consumption in mitochondria, is complex IV of the ETC, and one of the reversibly binding competitors to that site, is NO^●^ [15,16]. In that context, the continuous generation of NO^●^ from 2-signal activated macrophages maintaining a steady state concentration, is bound to and continuously inhibit electron transport and oxidative phosphorylation at the complex IV terminal point (heme a3-Cu_B_ center), where O_2_ is converted to H_2_O. Apart from inhibiting the respiration and hence, significantly decreasing the OCR of the activated macrophages, NO^●^ is a major consumer of O_2_ either for the formation of nitrate and nitrite, or the production of the deleterious peroxynitrite, through the rapid association with superoxide anion [17]. The latter is a product of electron leakage from the ETC and consequent reduction of the ambient oxygen by the “stray” electrons [10,18]. Electron transport chain complexes I and III have been traditionally identified as the main generators of superoxide anion in the mitochondria, most probably at their respective quinone-reducing centers, where the q10 semiquinone is more stably produced [10,19]. Nevertheless, other electron transporting enzymes are also responsible for superoxide production, both in the mitochondria [10] and in other cell compartments, like xanthine oxidase and NADPH oxidases, among others [20]. Interestingly enough, inhibition of complexes I, III or IV has been found to increase ROS formation [21]. In particular, inhibition of complex IV by 2 mM cyanide, resulted in a profound increase in ROS, leading to 57% inactivation of aconitase [21]. It is accordingly expected, that inhibition of complex IV by NO^●^ can both increase the generation of superoxide anion, and the formation of peroxynitrite, increasing significantly oxygen consumption. In the present work, we have seen that use of NO^●^ inhibitors and scavengers, just prior to, and during the course of the 2-signal activation of macrophages, have led to a partial and not complete abrogation of the OCR values. This is mainly evident through the maximal respiratory capacities of the macrophages in Figure 2C,D. Before treatment (untreated), the basal/maximal respiration ratio is ~8 while following iNOS inhibition by 1400 W or carboxyPTiO scavenging of NO^●^ the maximal respiration is only abrogated to a ratio of ~4 over basal respiration at best. This suggests that i) the iNOS inhibition or NO^●^ scavenging is not 100% and/or that ii) even though the NO^●^ inhibition of complex IV is reversible, there is a permanent damage to the respiratory chain enzymes [22]. Indeed it has been shown that peroxynitrite can react with mitochondrial membranes, causing significant inhibition to complexes I, II and V (50–80%) and to a lesser extend to complex IV [23]. The damage to the mitochondrial enzymes by the production of peroxynitrite has been linked with serious neuropathological implications [24], while peroxynitrite-mediated damage to the mitochondrial DNA has also been reported, leading to dose-dependent inhibition of mitochondrial protein synthesis [25]. Of course, as can be seen from the representative graphs in Figure 2C, scavenging of peroxynitrite by uric acid did not lead to a restitution of the maximal respiratory capacity, however and apart from the fact that the scavenging may have not been adequate, neither NO^●^ nor O_2_^●−^ are inhibited/scavenged by uric acid. In our hands, uric acid did not have the partial respiration-rescue effect, reported by Szabó and Salzman [26] on a macrophage cell line (J774.2) following 2-signal (IFNγ + LPS) activation. It has to be noted however that in our case the OCR measurements were performed by more sensitive techniques (Seahorse), given the fact that MTT is not exclusively reduced in the mitochondria [27]. Superoxide in physiological conditions may not be so damaging to the mitochondria *per se*, but this may not hold for its aberrant production. e.g., under inhibition. In addition, superoxide gets very rapidly dismutated to H_2_O_2_ by the cytosolic and mitochondrial superoxide dismutases (Cu-Zn and Mn SOD respectively) and in turn H_2_O_2_ can give very reactive hydroxyl radicals via Fenton reactions with transition metals (Cu, Fe). These reactive oxygen species can lead to lipid peroxidation of membranes, mitochondrial DNA damage and protein modification, resulting ultimately in mitochondrial dysfunction [28].

Despite the damage in the ETC chain following INFγ + LPS acivation, it can be seen from the representative data in Supplementary Figure S1, that basal glycolysis is not increased following the repression of respiration to compensate for the lost ATP production. Following the addition of oligomycin, the glycolytic capacity of macrophages may be compromised after the activation. Thus in our hands, M(2-signals) seem incapable of switching more to glycolysis to compensate for the loss of respiratory ATP. This might imply either a moderate damage to some enzymes or functions of the glycolytic pathway or some regulatory role of RNS. For example it has been shown that although NO^●^ inhibits respiration, at the same time it stimulates glucose-6-phosphate dehydrogenase, the first and rate-limiting step of the pentose–phosphate pathway [29]. Elsewhere, treatment of rat heart myoblasts and isolated mitochondria with peroxynitrite was found to cause the partial dissociation of HK II from mitochondria [30], while glyceraldehyde-3-phosphate dehydrogenase (GAPDH) was also found to be inhibited by peroxynitrite at a IC50 of 17 μM [31]. In parallel to RNS, ROS and oxidative insults can also modify key glycolytic proteins [32,33], or again by diverting glycolysis to the pentose-phosphate pathway to combat the oxidative stress [34]. Along the lines of ATP compensation by glycolysis, a possible alternative would be that M(2-signals) macrophages may have lower energy demands than their untreated counterparts. This in turn would mean that activated macrophages switch from performing basic, but highly ATP consuming, cellular processes, to more acute effector functions, reflecting thus the need for drastic response to potential threats in their microenvironment (e.g., pathogens). This is emphasized by our observation of inhibited proliferation in M(2-signals) macrophages (unpublished data).

In addition to these biological effects of RNS and ROS in the respiration at the cell level, we have revealed a cell-independent effect of NO^●^ and other ROS and RNS in extracellular oxygen consumption, using the Seahorse metabolic analysis. Using agents that can either produce NO^●^ (SNAP) or both NO^●^ and O_2_^●−^ (SIN-1), we demonstrated measurable oxygen consumption rates for both of these and their resulting species (ONOO^−^) in wells with media only, at physiological conditions and without any respiring entities (as demonstrated by the background ECAR). Of note, the OCR levels of SIN-1 were similar to those of M(2-signals)–in contrast to those of SNAP–during the whole period of the mitostress assay, presumably due to the additional production of O_2_^●−^, thus more closely resembling the cellular (activated macrophages) scenario. The NO^●^ and O_2_^●−^ production from the donors started when the microplates were equilibrated at 37 °C. This oxygen consumption was partly reversible with the use of NO^●^ and O_2_^●−^ scavengers (PTiO -data not shown- and MnTmPyP respectively). Furthermore, the use of MnTmPyP partly abrogated the oxygen consumption only in the SIN-1 and not in the SNAP wells, also pointing to the role of O_2_^●−^ in the oxygen consumption. The in situ addition of both SIN-1 and SNAP to naïve macrophages, immediately dropped their maximal respiratory capacity. Since the two donors were added just prior to the Seahorse measurements, it could be that this drop in the maximal respiratory capacity was simply due to NO^●^ inhibition of the respiration and not due to damage to the respiratory machinery, although this cannot be completely excluded. Moreover and although the extracellular NO^●^ donors effect on oxygen consumption is a possibility, the respiration profile of macrophages plus NO^●^ donors, differed greatly from that of cell-free SNAP or SIN-1, alluding to a rapid biological effect of NO^●^ donors in cellular OXPHOS.

**Figure 4:**
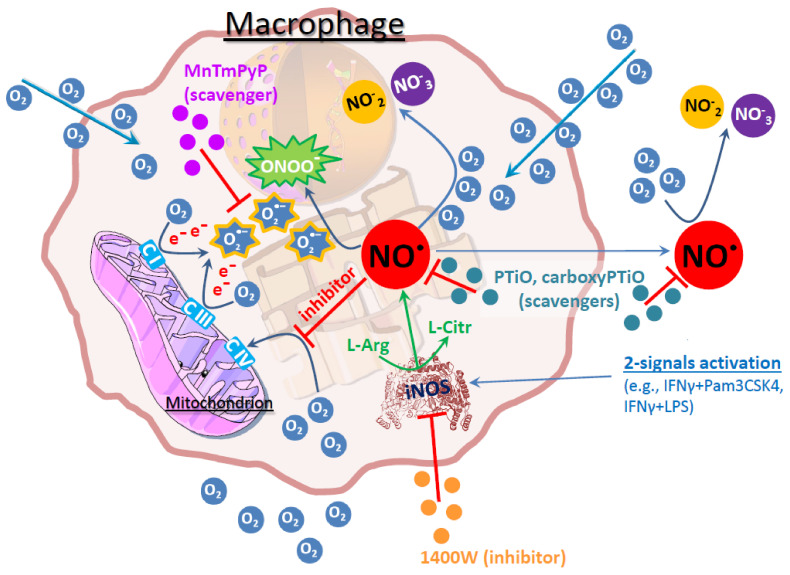
Overview of the processes affecting oxygen consumption in M(2-signals) macrophages. The main source of oxygen consumption in macrophages (as in all cells) is the terminal electron (e^−^) donor at ETC complex IV (heme a3-Cu_B_ center). Upon 2-signals activation (e.g., IFNγ + LPS or IFNγ + Pam3CSK4), the macrophages express iNOS, leading to production of NO^●^. NO^●^ reversibly inhibits the O_2_ attachment to complex IV, causing even higher electron leak from the ETC. These electrons can reduce O_2_ to O_2_^●−^, which then can bind with NO^●^ and produce ONOO^−^. Alternatively, NO^●^ can react with O_2_ and produce NO_2_¯ and/or NO_3_¯. Inhibitors [1400 W (iNOS)] and scavengers [PTiO/carboxyPTiO (NO^●^), MnTmPyP (O_2_^●−^)] on various levels (indicated with red lines) hamper the production of these free radicals, leading to the partial recovery of respiration.

In the present work, we have shown that the oxygen consumption of NO^●^ producing 2-signal activated macrophages does not reflect the actual respiratory function of the cells. The actual respiration is masked by an additional extracellular consumption of oxygen, which comes from the excessive production of free radicals intracellularly, and which eventually diffuse outside onto the bulk media surroundings (Figure 4). In that context our present work provide an additional explanation for the apparent unresponsiveness of the activated macrophages to external stimuli like the modulators in the seahorse “mitostress” assay, i.e., the sequential injections of oligomycin, FCCP and antimycin A/rotenone. The extracellular oxygen consumption is partly responsible for masking these responses which are already highly diminished from the respiratory inhibition. Even in cases where the injections-effects are still evident, both the basal and maximal OCRs following activation may not be what they appear. A steady state signal, corresponding to the amount of ROS and RNS extracellular consumption, has to be subtracted from the respiratory profile to reveal the real respiratory activity of the cells. However, given the inseparable biological role of ROS and RNS in macrophage activation, this is not practically feasible to do experimentally. Inhibition of iNOS prior to macrophage activation, partially reverses the tumoricidal properties of the cells, thus inhibits a full-extent activation, while inhibition of the reactive species following the activation has no effect in respiration of the activated macrophages (data not shown), probably due to the already induced mitochondrial damage downstream of NO^●^ production, during activation. Moreover, the effect of “pseudorespiration” in the bulk medium surrounding the cells should not be neglected, as one has to take into account that the seahorse OCR/ECAR measurements are conducted in a media microchamber of only 2.28 μL.

## 5. Conclusions

According to our findings presented here, the measured OCR by XFe Seahorse metabolic analyzers in intact cells excessively-producing reactive oxygen/nitrogen species (e.g., 2-signal activated M1 macrophages), does not directly correspond to their actual respiratory rate. There is also a non-respiratory oxygen consumption, derived from the cell-generated RNS and ROS which chemically deplete oxygen from the measurement micro-chamber. This component, which in some cases even masks the real respiratory profile of the cells, has to be taken into consideration and the composite OCR measurements have to be analyzed with caution.

## Data Availability

All data generated or analysed during this study are included in this published article and its Supplementary Information Files.

## Supplementary Materials

The following are available online at https://www.mdpi.com/article/10.3390/antiox10071149/s1, Figure S1. NO^●^ and O_2_^●−^ have no effect on the media acidification rates. Macrophages were activated for 24 h with the IFNγ + LPS (40 ng/mL and 10 ng/mL respectively) or not (untreated) and subjected to serial injections of oligomycin (2 μΜ), FCCP (1 μΜ) and antimycin A/rotentone (2/2 μΜ), according to the mitostress protocol. A Seahorse XFe96 metabolic analyzer was used to record the changes in the media acidification rate (ECAR). The reactive species-donors SIN-1 (NO^●^ and O_2_^●−^) or SNAP (NO^●^) were added (3mM for either) prior to the assay in cell-free, media-only wells. Horizontal intermittent line indicate the minimum (zero) ECAR values. The corresponding ECAR values of the experiment in Figure 1 are shown, with data presented as mean±STD of at least three replicates per condition. Any ECAR values following the addition of FCCP are not shown, due to the interference of H^+^ shuttled by the protonofore. Figure S2. O_2_^●−^ scavenging lowers the cell-free oxygen consumption rates. The reactive species-donors SIN-1 (NO^●^ and O_2_^●−^) or SNAP (NO^●^) were added (3mM for either) to cell free-wells with or without the addition of the superoxide scavenger MnTmPyP (15 μΜ). The Seahorse mitostress protocol was used to estimate the changes in the oxygen consumption rate (OCR). The lowest in value measurement following the addition of antimycin A/rotentone injection was used to calculate the differences in the oxygen consumption, following baseline subtraction. Horizontal intermittent line indicate the OCR levels of SIN-1. One representative experiment is shown, with data presented as mean ± STD of at least three replicates per condition.

## Abbreviations

BMDM	bone marrow derived macrophages
DAF-FM	4-Amino-5-methylamino-2′,7′-difluorescein
ECAR	extracellular acidification rate
FCCP	carbonyl cyanide-4-(trifluoromethoxy)phenylhydrazone
IFN	interferon
iNOS	inducible nitric oxide synthase
LPS	lipopolysaccharide
NO^●^	nitric oxide
OCR	oxygen consumption rate
OXPHOS	oxidative phosphorylation
RNS	reactive nitrogen species
ROS	reactive oxygen species
SIN-1	3-morpholinosydnonimine
SNAP	S-nitroso-N-acetyl-DL-penicillamine
SRC	spare respiratory capacity
2-DG	2-deoxy-d-glucose
