# Supplementary figures and images for: Reactive Species from Two-Signal Activated Macrophages Interfere with Their Oxygen Consumption Measurements
Source: Antioxidants (Basel). 2021 Jul 20;10(7):1149. doi: 10.3390/antiox10071149 (PMC8301004; doi:10.3390/antiox10071149)

Supplementary Figure S1

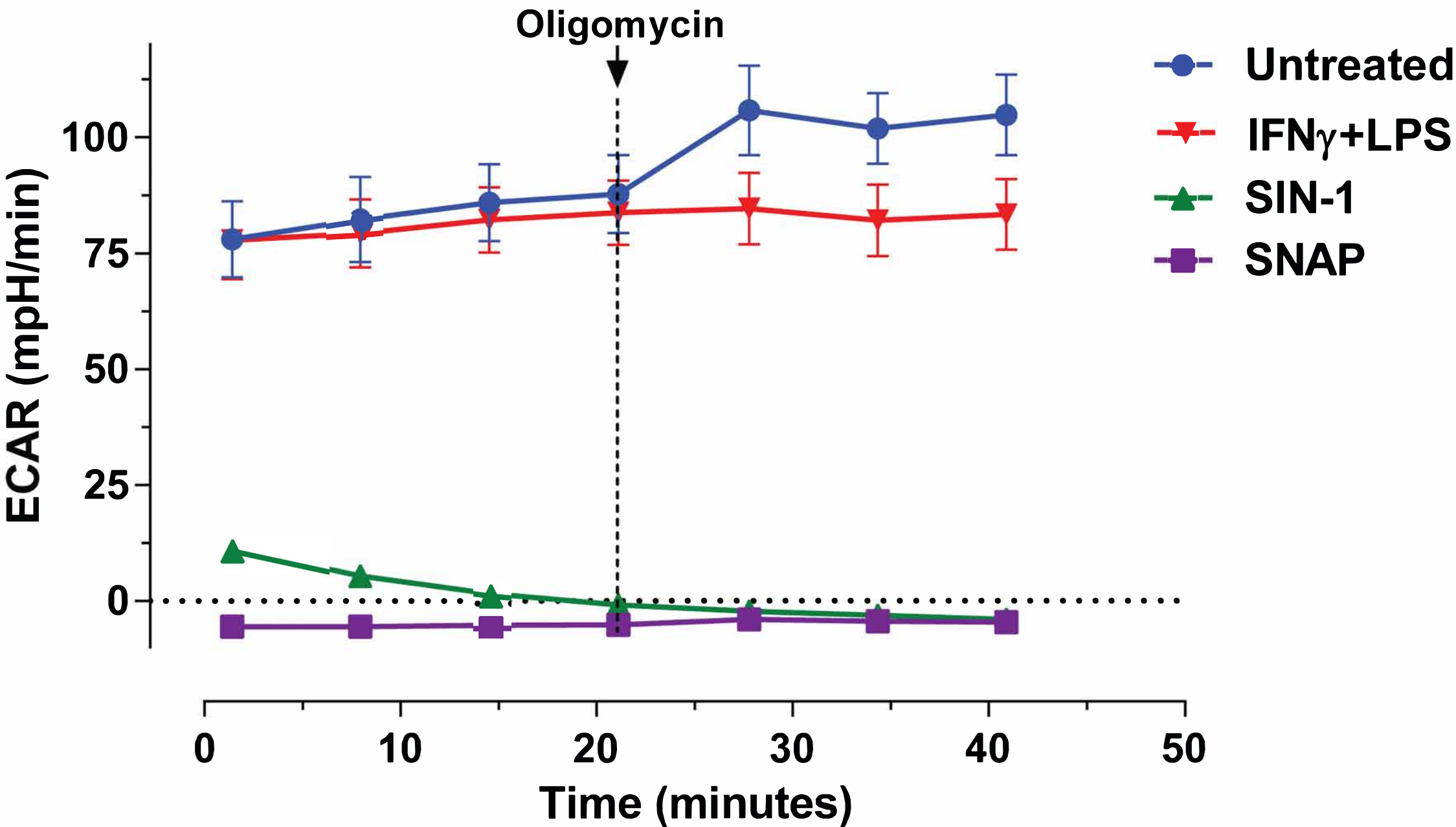

**Supplementary Figure S2**

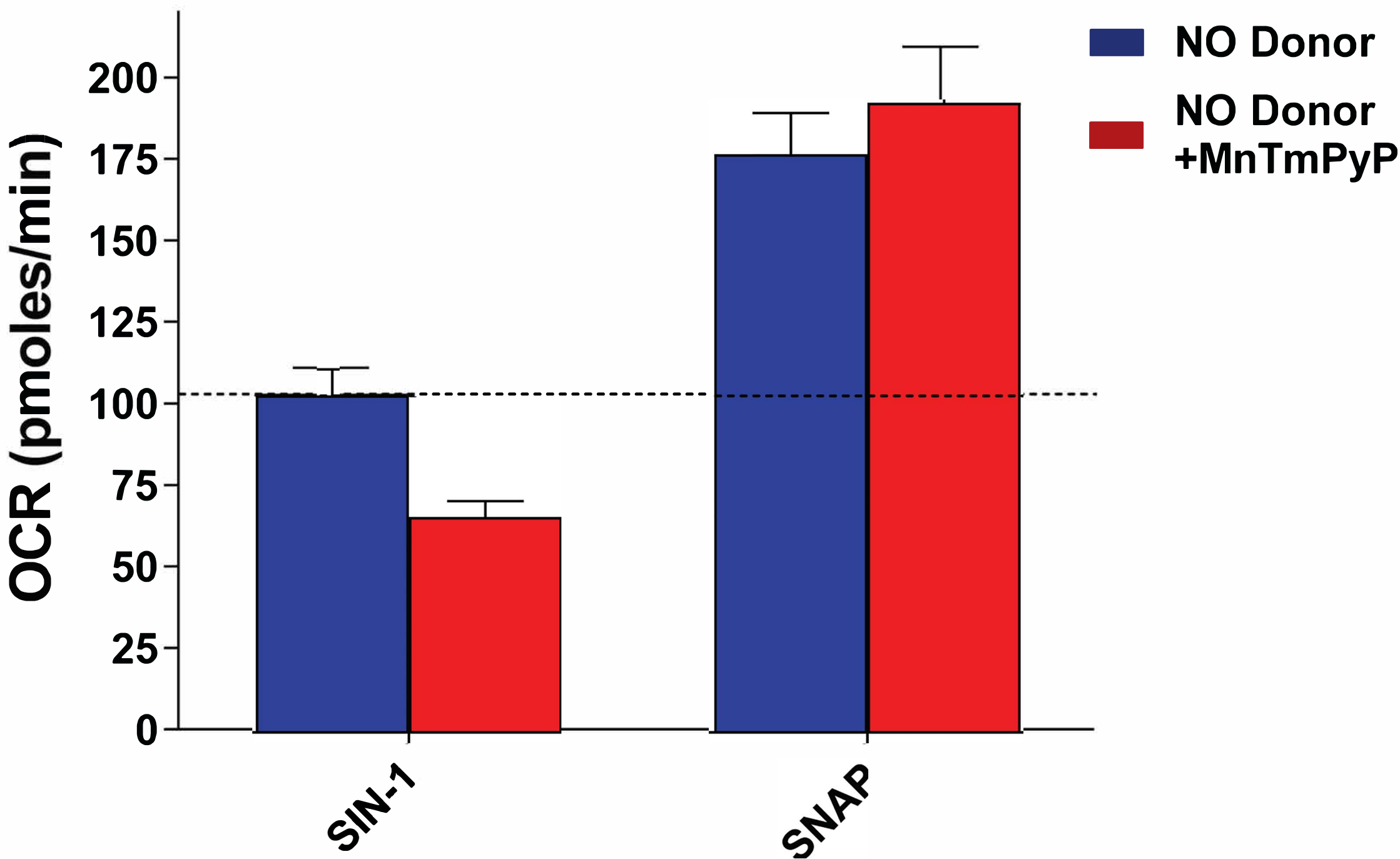

Supplement: Supplementary file 1 [file antioxidants-10-01149-s001.zip › antioxidants-1275933-supplementary.pdf]
